# 7**α**-Hydroxy-**β**-Sitosterol from *Chisocheton tomentosus* Induces Apoptosis via Dysregulation of Cellular Bax/Bcl-2 Ratio and Cell Cycle Arrest by Downregulating ERK1/2 Activation

**DOI:** 10.1155/2012/765316

**Published:** 2012-09-11

**Authors:** Mohammad Tasyriq, Ibrahim A. Najmuldeen, Lionel L. A. In, Khalit Mohamad, Khalijah Awang, Noor Hasima

**Affiliations:** ^1^Genetics & Molecular Biology Division, Institute of Biological Science, Faculty of Science, University of Malaya, 50603 Kuala Lumpur, Malaysia; ^2^Chemistry Department, Faculty of Science, University of Zakho, Zakho, Kurdistan Region, Iraq; ^3^Department of Pharmacy, Faculty of Medicine, University of Malaya, 50603 Kuala Lumpur, Malaysia; ^4^Centre for Natural Product Research and Drug Discovery (CENAR), Department of Chemistry, Faculty of Science, University of Malaya, 50603 Kuala Lumpur, Malaysia

## Abstract

In continuation of our interest towards the elucidation of apoptotic pathways of cytotoxic phytocompounds, we have embarked upon a study on the anticancer effects of 7**α**-hydroxy-**β**-sitosterol (CT1), a rare natural phytosterol oxide isolated from *Chisocheton tomentosus*. CT1 was found to be cytotoxic on three different human tumor cell lines with minimal effects on normal cell controls, where cell viability levels were maintained ≥80% upon treatment. Our results showed that cell death in MCF-7 breast tumor cells was achieved through the induction of apoptosis via downregulation of the ERK1/2 signaling pathway. CT1 was also found to increase proapoptotic Bax protein levels, while decreasing anti-apoptotic Bcl-2 protein levels, suggesting the involvement of the intrinsic pathway. Reduced levels of initiator procaspase-9 and executioner procaspase-3 were also observed following CT1 exposure, confirming the involvement of cytochrome c-mediated apoptosis via the mitochondrial pathway. These results demonstrated the cytotoxic and apoptotic ability of 7**α**-hydroxy-**β**-sitosterol and suggest its potential anti-cancer use particularly on breast adenocarcinoma cells.

## 1. Introduction

The tropical plant* Chisocheton tomentosus *from the Meliaceae family is a medium-sized tree that can grow upto 21 m in height [1]. *Chisocheton* species were known to produce bioactive compounds with complex molecular structures such as erythrocarpine E and chisomecine A [2, 3]. Plants from this family have been known to be a rich source of secondary metabolites including various sterols, terpenoids, and alkaloids, with medicinal and pesticidal properties such as antifungal, antibacterial, antiviral, anti-inflammatory, and antiplasmodial agents [4–6]. In tropical countries, this plant has been used as a form of traditional medicine against several diseases including diabetes, malaria, liver, and cancer diseases [7–9].

Plant-derived sterols or phytosterols are structurally similar to cholesterol with a slight difference at the C-24 position containing an additional ethyl group [10]. About 44 phytosterols have been identified to date, with major forms existing in higher plants constituted by **β**-sitosterol, campesterol, and stigmasterol [11–14]. Past studies have also shown that **β**-sitosterol possesses a relatively large dipole moment, giving it a polar or hydrophilic nature which is a desirable trait in most *in vivo* drug applications [13]. **β**-sitosterols have also been reported to bind various carrier proteins such as human serum albumin mainly by hydrophobic and hydrogen bond interactions, thus making protein-drug combination complexes a viable option for chemotherapy [15].

Sitosterols often undergo oxidation processes to form sitosterol oxidation products (SOPs) [16]. These forms of sterols are structurally different from parent with an additional steroid ring group of either hydroxy- (OH–), keto (=O), epoxy, or triol [17]. Various forms of SOP that exist in human plasma are **β**-epoxysitostanol, sitostanetriol, campestanetriol, **α**-epoxysitostanol, 7-ketositosterol, and 7**β**-hydroxysitosterol, with **β**-epoxysitostanol, sitostanetriol, and campestanetriol being the most abundant SOPs [18].

In recent years, researchers have been focusing on the effects of phytosterols toward various cancer cell lines and their implication in multidrug resistance [19–21]. To date, several apoptotic pathways mediated by phytosterols have been proposed in cancer culture models such as the Ras/ERK and the PI3 K/Akt pathway [22–26]. In contrast, investigation on the mechanistic effects of phytosterol oxides is still scarce. Despite several reports on the cytotoxicity of these compounds, their cytotoxic mechanism of action is, however, still unclear [18, 27]. Roussi et al. [28] suggested that 7**β**-hydroxysitosterol, one of the many phytosterol oxides, could target the mitochondria, leading to loss of mitochondrial membrane potential to induce cytochrome c release. However, the mechanism of cytochrome c release and caspase activation was not well characterized [28–30].

In this study, the cytotoxic effects of 7**α**-hydroxy-**β**-sitosterol (CT1) isolated from *Chisocheton tomentosus* (Meliaceae) was investigated on human breast, liver, and oral cancer cell lines, while its apoptotic potential and anticancer mechanism was elucidated on MCF-7 human breast cancer cell line for the first time.

## 2. Materials and Methods

### 2.1. Plant Material

Dried bark of* Chisocheton tomentosus* was collected from Mersing, Johor, Malaysia in 1993. The sample was identified by Mr. Teo from the Department of Chemistry, Faculty of Science, University of Malaya. A voucher specimen KL-4251 was deposited in the Department of Chemistry Herbarium, University of Malaya.

### 2.2. Reagents

Dulbecco's modified Eagle's medium (DMEM) and Roswell Park Memorial Institute-1640 (RPMI-1640) were purchased from Thermo Scientific (IL, USA). Trypsin and fetal bovine serum (FBS) were purchased from Sigma Aldrich (KS, USA). Mammary epithelial growth media (MEGM) and all antibiotics were purchased from Lonza Inc. (MD, USA). Dimethyl-2-thiazolyl-2,5-diphenyl-2H-tetrazolium bromide (MTT) reagent, annexin V-fluorescein isothiocyanate (FITC) apoptosis detection kit, propidium iodide (PI), RNase and SuicideTrack DNA ladder isolation kit were purchased from Calbiochem (CA, USA). Primary antibodies against caspase-9, caspase 3, caspase-6, caspase-8, XIAP, Bcl-2, Bax, Bim, Fas-L, p42/44, and *β*-actin were obtained from Cell Signaling (MA, USA).

### 2.3. Extraction and Isolation Compounds

Dried ground bark of *Chisocheton tomentosus* (3.5 kg) was first defatted with hexane for five days, followed by dichloromethane (DCM) for five days. DCM was removed through evaporation using a rotary evaporator. The crude extract (10.0 g) was subjected to a silica gel column (hexane : DCM, 95 : 5) and eluted gradiently using hexane : DCM and DCM : acetone to yield five fractions. The fifth fraction (DCM : acetone, 60 : 40) was further subjected to an isocratic separation using a silica gel column with (acetone : DCM : hexane, 25 : 25 : 50) to give five other fractions. The third fraction in the solvent was allowed to evaporate to yield colorless crystals of 7**α**-hydroxy-**β**-sitosterol or CT1 (0.2 g). Identification of CT1 was elucidated by comparison with known samples by their UV, IR, 1D-NMR, and 2D-NMR spectra and melting points [31]. A complete description of the entire extraction process and identified compounds is available under Supplementary Material  1 (see Supplementary Material available online at doi: 10.1155/2012/765316).

### 2.4. Cell Lines and Culture Conditions

A set of four human tumor cell lines were used in this study: Ca Ski cervical cancer cells and HepG2 liver cancer cells which was obtained from Professor Dr. Rohana, University of Malaya Medical Center (UMMC), HSC-4 oral cancer cells and MCF-7 breast adenocarcinoma cells were obtained from Dr. Eswary Thirthagiri, Cancer Research Initiative Foundation (CARIF, Malaysia), and HMEC human mammary epithelial cells purchased from Lonza Inc. (MD, USA) was used as a normal cell control. For routine maintenance, MCF-7 cells were cultured in RPMI-1640, while HSC-4, Ca Ski, and HepG2 cells were cultured in DMEM, with both media types supplemented with 10% (v/v) FBS, 100 U/mL penicillin, and 100 mg/mL streptomycin. HMEC cells were cultured in serum-free mammary epithelial growth media (MEGM). Cells were grown as monolayers at 37°C in humidified atmosphere with 5% CO_2_ and 95% air.

### 2.5. MTT Cell Viability Assay

Cell viability was measured using the MTT assay method. Briefly, CT1 was dissolved in dimethyl sulfoxide (DMSO) to a final concentration of 10.0 mM. Cells were seeded at 1.0 × 10^4^ cells/well in 96-well plates and treated with final CT1 concentrations from 0.5 to 80.0 *μ*M for 24 h with DMSO as a solvent control. After treatment, 10.0 *μ*L MTT (5.0 mg/mL) was added to each well and incubated at 37°C for 2 h. After incubation, the medium was replaced with 200.0 *μ*L of DMSO and absorbance measured at 570 nm for each well using a microplate reader (Tecan Sunrise, Switzerland). Absorbance was compared against vehicle-treated control and expressed as mean percentage of viable cells.

### 2.6. Live/Dead Assay

Cytotoxic effects of four cell lines treated with CT1 were performed using LIVE/DEAD viability/cytotoxicity kit for mammalian cells (Invitrogen, NY, USA) according to manufacturer's protocol. Briefly, cells were grown on cover slips and treated for 12 h. Staining was done using a 150.0 *μ*L dual fluorescence staining system consisting of calcein acetoxymethyl ester (2.0 *μ*M) and ethidium homodimer (4.0 *μ*M). Excitation and emission wavelengths of both fluoresceins were set at 494/517 nm for calcein acetoxymethyl ester and 528/617 nm for ethidium homodimer, respectively. Samples images were capture using a Nikon Eclipse TS-100 fluorescence microscope (Nikon, Tokyo, Japan) under 100x magnification. All images were analyzed using the NIS Element D 3.1 data analysis software (Nikon, Tokyo, Japan).

### 2.7. Wound Healing Assay

Wound healing was used to examine the antimigration effects of CT1 on cancer cells. Briefly, cells were grown on 6-well plates for 24 h followed by treatment with mitomycin-c for 2 h. Media was removed and equal size wounds were created with a pipette tip. Cells were washed twice with 1x PBS and serum free media was added. Each group was incubated with media only, DMSO, or CT1 for 24 h. Images were captured using a Nikon Eclipse TS-100 fluorescence microscope (Nikon, Tokyo, Japan) under 100x magnification and analyzed using T-Scratch software v7.8 [32].

### 2.8. Cell Cycle Analysis

Both MCF-7 and HMEC cells were treated with CT1 for 12 h and 24 h before fixation. Briefly, 1.0 × 10^5^cells was pelleted, rinsed with 1x PBS, and fixed with 70% (v/v) cold ethanol while mixing. Fixed samples were stored at −20°C overnight and stained with 1.0 mL of PBS containing 50.0 *μ*g/mL PI and 0.1 mg/mL RNase A. Fluorescence from a population of 1.0 × 10^4^ cells were detected using the FASCanto II (Becton Dickinson, CA, USA) and analyzed using the ModFit Lt 3.2 software (Tree Star Inc, OR, USA).

### 2.9. Annexin V-FITC/PI Analyses

Detection of apoptosis was conducted using the Annexin V-FITC/PI apoptosis detection kit according to manufacturer's protocol. Briefly, both CT1-treated and untreated MCF-7 and HMEC cells were washed twice with 1x PBS and resuspended in binding buffer. Cell suspensions were stained with 1.25 *μ*L of annexin V-FITC conjugate and 10.0 *μ*L of PI. Detection of signals from 1.0 × 10^4^ cells were obtained using the FASCanto II (Becton Dickinson, CA, USA) and analyzed using the FACS Diva acquisition and analysis software (Becton Dickinson, CA, USA).

### 2.10. DNA Fragmentation Assay

Detection of apoptotic fragmented DNA was performed using the Suicide-Track DNA isolation kit according to manufacturer's protocol. After 24 h of incubation with CT1 cells were collected and total DNA was extracted from cells. Samples were loaded into a 1.5% (w/v) agarose gel and separated by electrophoresis. DNA fragments were stained with ethidium bromide and were visualized under UV illumination using the Alpha Imager Gel Documentation System 2000 (Inotech, CA, USA).

### 2.11. Western Blot

To determine pro- and antiapoptotic protein levels, CT1-treated MCF-7 total proteins were extracted using the NE-PER nuclear and cytoplasmic extraction kit (Pierce, IL, USA) according to manufacturer's protocol. Protein concentrations were determined using the Bio-Rad DC protein assay (Bio Rad, CA, USA) according to manufacturer's protocol. Equal amounts of protein were subjected to 12% to 15% (w/v) SDS-PAGE and transferred onto 0.2 *μ*M nitrocellulose membranes using a Trans Blot Semidry blotter (Bio Rad, CA, USA). Membranes were blocked either with 2% (w/v) BSA or 2% (w/v) nonfat skim dry milk, and incubated with primary antibodies against *β*-actin, Bax, Bcl-2, Bim, caspase-6, caspase-3, caspase-9, caspase-8, XIAP, FasL, and ERK1/2. Horseradish peroxidase-(HRP-)linked secondary antibodies were added and bound proteins were detected through enhanced chemiluminescence reagent exposed on X-ray films. Relative intensities of bands were quantified using the ImageJ v1.43 analysis software (NIH, MD, USA).

### 2.12. Statistical Analysis

All results were expressed as mean ± SEM obtained from three independent experiments. Statistical significances between various groups were determined using one-way ANOVA with a *P* value ≤0.05 threshold.

## 3. Results and Discussion

### 3.1. Characterization of 7*α*-Hydroxy-*β*-Sitosterol (CT1)

**Figure 1:**
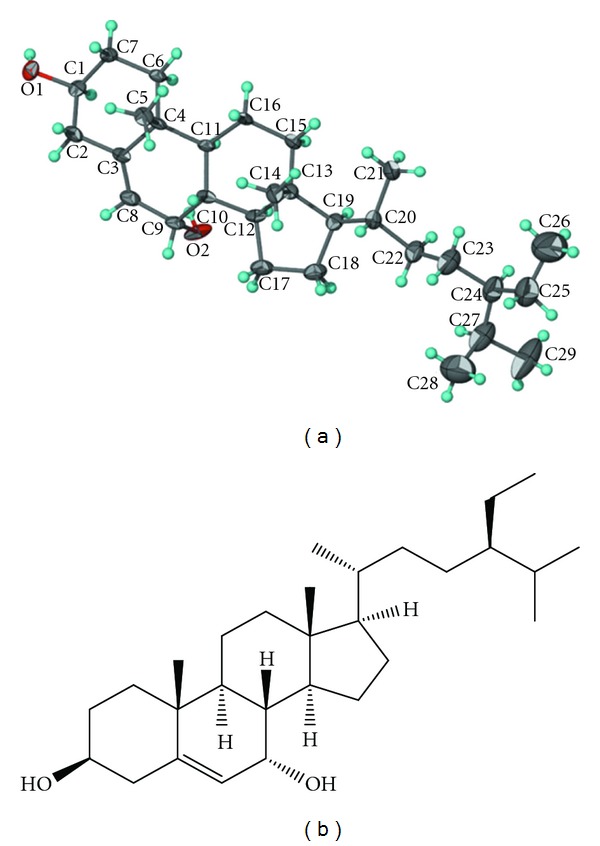
(a) Molecular structure of 7**α**-hydroxy-**β**-sitosterol (CT1). The ellipsoids denote 70% probability. Hydrogen atoms are drawn as spheres of arbitrary radii [33]. (b) Chemical structure of 7**α**-hydroxy-*β*-sitosterol isolated from *Chisocheton tomentosus* bark.

CT1 was isolated as a colorless crystal with the UV spectrum showing absorption at *λ*_max⁡_ 302 nm and 254 nm, IR spectrum showing the presence of hydroxyl group by the absorption band at *ν*_max⁡_ 3430 cm^−1^, and GCMS spectrum revealing a molecular ion peak [M]^+^ at m/z 430, corresponding to a molecular formula of C_29_H_50_O_2_. The ^1^H-NMR spectrum showed six methyl groups resonating as singlets, doublets, and triplets. Broadband decoupled ^13^C-NMR spectrum displayed twenty-nine carbon atoms in the molecule, the DEPT spectra exhibited six methyl, ten methylene, and ten methine carbons, while the remaining three carbons were quaternary as deduced from broadband spectrum. All protons of methyl, methylene, sp^3^ methine, and sp^2^ methine were approved by HMQC. HMBC spectrum showed long range correlation of C-19 proton with quaternary carbon of C-10, while C-18 methyl protons showed HMBC connectivity with quaternary C-13, indicating that the methyl group (19 and 18) was attached directly with C-10 and C-13, respectively, and the long chain substituent should be attached in the position of C-17 (Table 1). The signal C-5 (*δ*_c_ 146.3) of the compound was shifted toward the lower magnetic field (Figure 1(a)) [33]. Therefore, the configuration of hydroxyl group at C-7 was identified as an **α** form [34–37]. Consequently, CT1 was identified as 7**α**-hydroxy-**β**-sitosterol. (Figure 1(b)).

### 3.2. CT1 Displayed Cytotoxic Effects on Various Cancer Cell Lines

**Figure 2:**
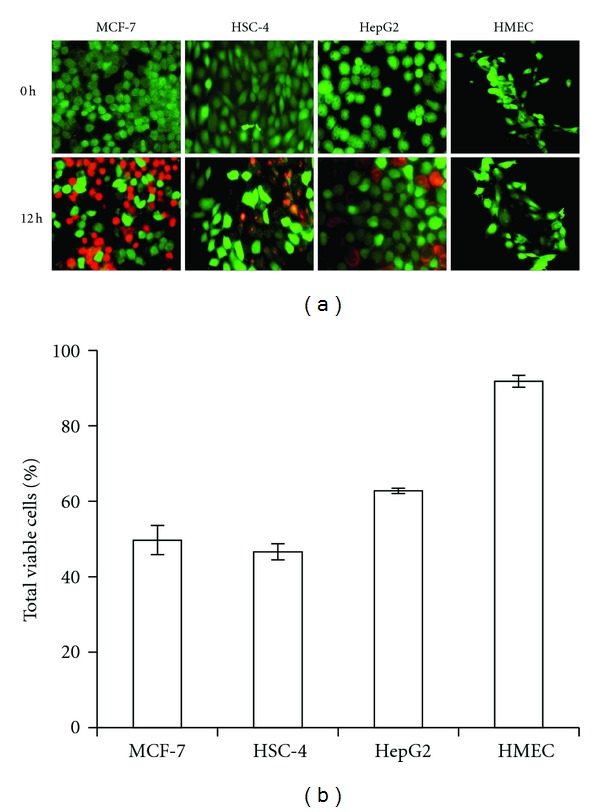
Live/dead viability/cytotoxicity assay depicting the cytotoxic effects of CT1 in tumor cell lines with minimal cytotoxic effects on HMEC normal cell controls. (a) Fluorescence microscope images of viable cells stained with acetomethoxy derivate of calcein (green) and nonviable cells stained with ethidium homodimer 1 (red). (b) Percentage of viable cells as calculated under a fluorescence microscope. A total of four random quadrants were selected from each triplicate for quantification. All data were presented as mean ± SEM.

MTT cell viability assay was used to evaluate the cytotoxic effects of CT1 on four human cancer and an HMEC normal cell line. Results indicated the induction of cytotoxicity in a dose- and time-dependent manner over 24 h of exposure. Minimal cytotoxic effects were observed on HMEC control cells, where 17.6 ± 4.2% killing was observed under similar treatment conditions, as opposed to 78.7 ± 3.1% in MCF-7 cells which indicated the lowest IC_50_ value of 16.0 ± 3.6 *μ*M among all cell lines tested (Table 2). Viability of cells treated with DMSO (≤2.0% v/v) was insignificantly affected (data not shown), thereby ruling out the occurrence of solvent-induced cytotoxicity. Live/dead viability/cytotoxicity assays (Figures 2(a) and 2(b)) corresponded with MTT data, thereby supporting the need to further investigate the apoptotic effects of CT1 for the treatment of breast cancer. All subsequent experiments were carried out based on IC_50_ values as obtained from MTT experiments.

### 3.3. CT1 Induces Apoptosis-Mediated Cell Death

**Figure 3:**
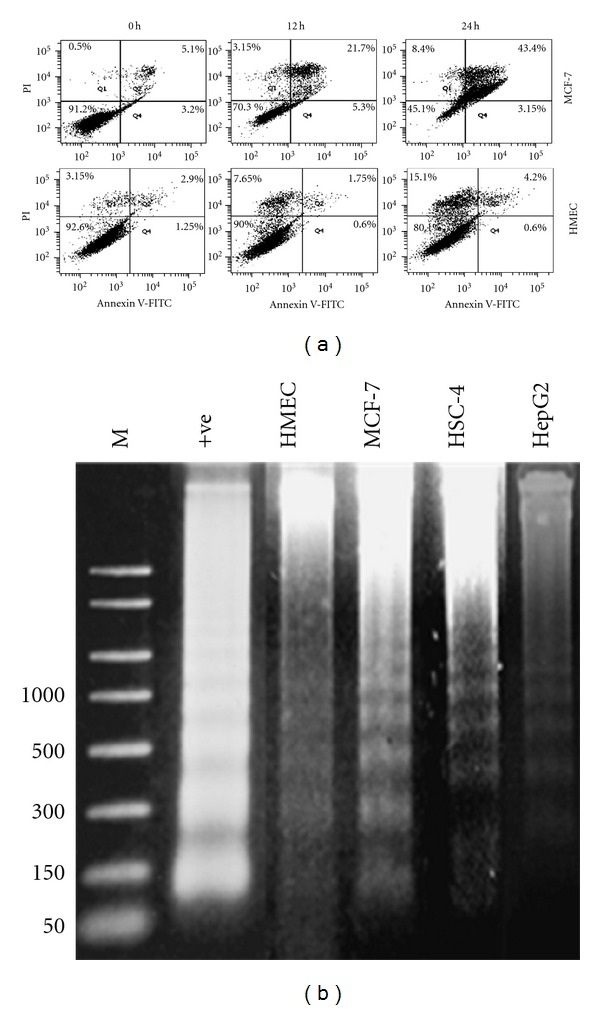
CT1 potentiates apoptosis-mediated cell death in MCF-7 human breast cancer cells. (a) Detection of apoptosis using flow cytometry after annexin V-FITC/propidium iodide (PI) staining. MCF-7 cells and HMEC cells were treated with CT1 at IC_50_ concentrations for 24 h. Dot plots are a representative of 1.0 × 10^4^ cells of three replicates with percentage of cells indicated in each quadrant. (b) Confirmation of apoptosis-mediated cell death through observation of a 200 to 250 bp DNA laddering using the DNA fragmentation assay. Cells were treated with CT1 for 24 h followed by analysis of extracted DNA on 1.0% (w/v) agarose gel electrophoresis. +ve: positive control. M: 100 bp DNA size marker.

We next determined whether CT1 cytotoxic effects were mediated through apoptosis or necrosis using DNA fragmentation assay and double fluorescence staining of annexin V-FITC/PI flow cytometry assay. An increase in cellular staining with FITC-conjugated annexin-V serves as an early marker for apoptosis while staining with PI indicates loss of cell membrane integrity. Treatment of MCF-7 cells was found to induce apoptotic cell death by observing a shift in viable cell population from early to late stage of apoptosis, followed by secondary necrosis. The percentage of MCF-7 viable cells decreased from 91.2% to 45.1%, while total percentage of apoptotic MCF-7 cells was 46.6% after 24 h (Figure 3(a)). Minimal apoptotic population shifts were observed in HMEC cells after similar treatments. DNA extracted from CT1-treated cells also showed DNA laddering with approximately 200 bp to 250 bp intervals as the result of endonuclease action at sites between nucleosomes, thus confirming the occurrence of apoptosis (Figure 3(b)).

### 3.4. Induction of Cell Cycle Arrest and Antimigration Effects of CT1

**Figure 4:**
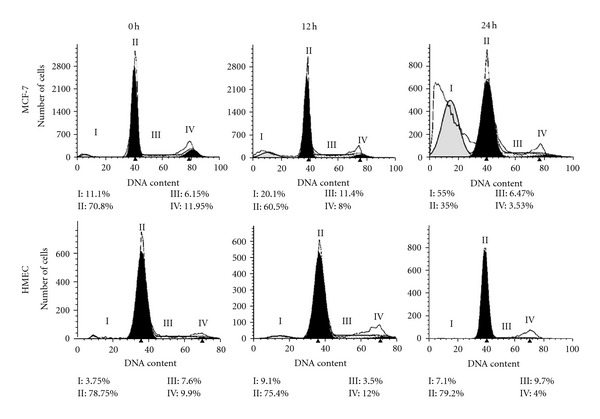
Cell cycle distribution of MCF-7 and HMEC cells using flow cytometry after staining with PI for 12 h and 24 h. I: Sub-G_1_; II: G_0_/G_1_; III: S; IV: G_2_/M. All experiments were a representative of 1.0 × 10^4^ cells and the percentage of cells in phases is indicated.

**Figure 5:**
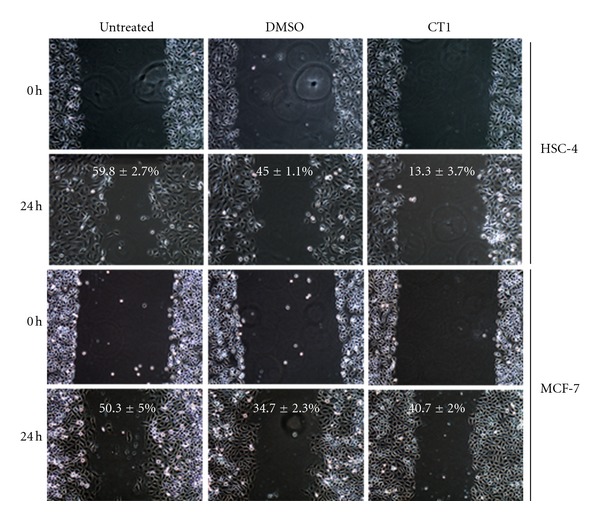
Wound healing assay displaying the antimigration effects of CT1 on HSC-4 cells, with minimal effects on MCF-7 cells. All cells were treated with mitomycin c to halt proliferation, followed by CT1 at IC_50_ concentrations for 12 h. Wound edge images of each independent triplicate were captured and measured at 24 h aftertreatment using T-scratch software, and percentage of migration is indicated as mean ± SEM.

The effects of CT1 on cell cycle progression using flow cytometry PI-based staining showed an increase in the population of sub-G_1_ phase from 11.1% to 55.0% after 24 h incubation. The increase was consistent with a reduction in the G_0_/G_1_ phase from 70.8% to 35.0% after 24 h indicating a potential cell cycle arrest during the G_0_/G_1_ phase as there were minimal changes in both S and G_2_/M phases after 24 h of CT1 treatment (Figure 4). Cell cycle profiles for HMEC normal human breast cell controls were found to be consistent after 12 h and 24 h exposure. Wound healing assays also showed that HSC-4 cells treated with CT1 migrated at a slower rate compared to MCF-7 cells, indicating that CT1 was more effective in preventing cellular migration in oral cancer (Figure 5). Antimigration effects on HepG2 cells were found to be absent with treated cells showing comparable migration rates as untreated cells (data not shown).

### 3.5. CT1 Reduces ERK1/2, FasL, Bcl-2, and Bim Protein Levels

**Figure 6:**
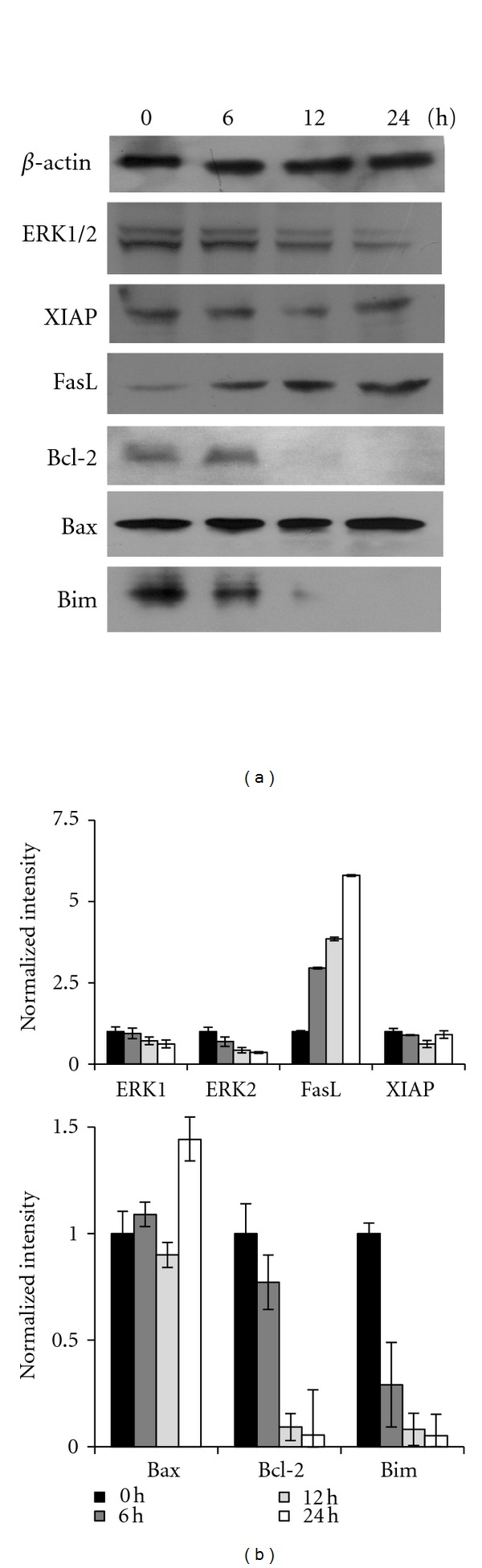
Observation on the effects of CT1 treatment over 24 h on MCF-7 protein levels using Western blot. (a) CT1 was found to decrease ERK1/2 and anti-apoptotic Bcl-2 and Bim protein levels, while increasing FasL protein levels. XIAP and proapoptotic Bax protein levels were unaffected following CT1 exposure. *β*-actin was used as a normalization control for all experiments. (b) Quantification of protein band intensities were determined by densitometry analysis and normalized to *β*-actin using the ImageJ v1.43 software. All results were presented as mean normalized intensity ± SEM of three independent experiments.

Western blot analysis of CT1-treated MCF-7 cells was carried out to observe the effects on ERK1/2, FasL, and Bcl-2 family of apoptotic proteins. Results showed that protein levels of both ERK1 and ERK2 declined over 24 h, while the level of the FasL increased dramatically by almost five-folds over 24 h compared to untreated cells (Figures 6(a) and 6(b)). Protein levels of the pro-apoptotic Bim and Bcl-2 also decreased after CT1 treatment and were completely absent 12 h aftertreatment, favoring the induction of apoptosis (Figures 6(a) and 6(b)). Furthermore, protein levels of pro-apoptotic Bax were found to be slightly elevated after 24 h, with consistent XIAP protein levels. Due to the dimerization nature of Bcl-2 family proteins, the ratio between Bax/Bcl-2 protein levels were measured and indicated a 9.7-fold increase at 12 h, which further increased to 26.6-folds higher compared to control cells at 24 h (Figures 6(a) and 6(b)).

### 3.6. CT1 Induces Intrinsic Caspase-Mediated Apoptosis in MCF-7 Cells

**Figure 7:**
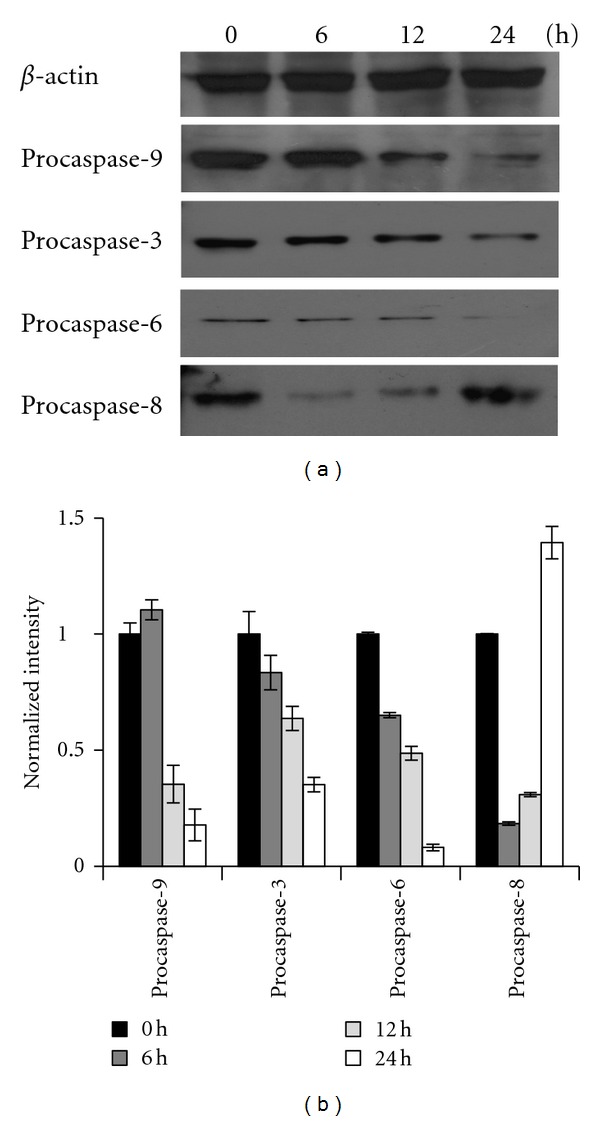
Activation of caspases upon CT1 treatment in MCF-7 cells. (a) Western blot analysis on protein levels of various procaspases upon CT1 treatment. MCF-7 cells was treated with 16.0 *μ*M of CT1 for 6 h, 12 h, and 24 h, respectively. Western blot of cell extracts were probed using the indicated procaspase antibodies and *β*-actin as a normalization control. (b) Normalization on band intensities between procaspases and *β*-actin was determined by densitometry using the ImageJ v1.43 software, and results were presented as a mean normalized intensity ± SEM of three independent experiments.

The involvement of FasL and Bcl-2 family members implied that induction of apoptosis by CT1 was mediated via both the mitochondrial and death receptor pathway. Therefore, we also assessed the protein levels of various procaspases through Western blot analysis. Our data demonstrated that CT1 induced the reduction of procaspase-3, -6, and -9 levels leading to an increase in active effector caspase forms (Figures 7(a) and 7(b)). Reduction in procaspase-8 protein levels was found to be transient over the first 6 h with protein levels starting to increase 12 h aftertreatment onwards, suggesting that the extrinsic death receptor pathway was used to augment the apoptotic signals arising from the intrinsic pathway.

## 4. Discussion

To date, natural plant compounds have shown great potential as apoptosis-inducing agents in cancer cells [38]. Results from this study clearly indicated the potential benefits of phytosterol oxides, specifically 7**α**-hydroxy-**β**-sitosterol in the prevention of breast adenocarcinoma. Current cytotoxicity data have demonstrated that IC_50_ values of CT1 after 24 h of incubation on all cancer cell lines tested were within the range of 16.0 *μ*M to 32.0 *μ*M, which is comparable to most commercialized phytocompounds. It is suggested that variations in CT1 toxicity in different cancer types may be attributed to various reasons including cellular accessibility, genetic variability of cell lines, and circadian variations [39]. In terms of structure-activity relationship, we found that the presence of a 7*α*-OH on CT1 augmented its cytotoxicity towards HepG2 cancer cells when compared to 7*β*-OH sitosterols [40]. Sitosterol derivatives with an oxidized steroid ring were also found to be a more favorable trait compared to oxidized stigmasterol derivatives following reports on cytotoxicity levels in MCF7 and HepG2 cells [10, 41]. These observations highlighted the importance of 7*α*-OH phytosterols in governing *in vitro* biological activity and suggested the superiority of CT1 (7**α**-hydroxy-**β**-sitosterol) over other phytosterol counterparts.

One of the crucial factors which contribute towards proliferating breast cancer cells is the activation of the mitogen-activated protein kinase (MAPK/ERK) cascade, which can be prevented by using agents that prevent ERK1/2 activation [42]. Extracellular signal-regulated kinase (ERK1/2), which is expressed ubiquitously in mammalian cells, is multifunctional serine/threonine kinases that phosphorylate a vast array of substrates localized in all cellular compartments [43]. In normal cells, sustained activation of ERK1/2 promotes G_1_ to S phase progression and inhibits antiproliferative genes, while its hypoactivation by MEK inhibitors can induce cells to undergo cell cycle arrest [44]. Our current study showed that CT1 was able to inhibit cell cycle progression at the G_0_/G_1_ phase after 24 h, which corresponded to reduced protein levels of ERK1/2 as shown in our Western blot analysis.

Members of the Bcl-2 family have been identified as key regulators of apoptosis and are further divided into pro-apoptotic and anti-apoptotic members [45]. Bcl-2 proteins often form heterodimer complexes with Bax proteins, which result in the release of cytochrome c from the mitochondria and subsequent induction of cell death [46]. Hence, an increase in the ratio of Bax/Bcl-2 is considered as one of the major markers of pre-apoptosis. Several natural compounds that have been shown to influence the Bax/Bcl-2 ratio in cancer cells are (−)-epigallocatechin-3-gallate (EGCG) and resveratrol [47, 48]. In reference to this, results of our study also suggest that CT1 effectively induces apoptosis in MCF-7 cells through dysregulation of the Bax/Bcl-2 ratio, which in turn results in the cleavage of procaspase-9, -3, and -6 into active effector caspases.

Western blot analysis also showed that treatment of CT1 not only activates apoptosis effector proteins such as caspase-3 and caspase-6, which is evident through a decrease in procaspase-3 and procaspase-6 protein levels, but also activates caspase-8. This strongly suggests that signals favoring the induction of apoptosis do not solely originate from the mitochondrial pathway, but may also be augmented by the extrinsic death receptor pathway, specifically through Fas-mediated mechanisms [22]. This is further supported in past studies revolving caspase cascades indicating that activated caspase-6 can directly process the transient activation of caspase-8 through an amplification loop to enhance apoptotic signals [49]. In agreement with this, we observed an initial reduction in procaspase-8 levels at 6 h, followed by a subsequent increase over the next 12 h.

## 5. Conclusion

For the first time, the biological activity of the active phytocompound, 7**α**-hydroxy-**β**-sitosterol (CT1) isolated from *Chisocheton tomentosus*, was characterized in various cancer cell lines. This compound was found to significantly inhibit the proliferation of MCF-7 human breast cancer cells among other cancer cell lines through dysregulation of Bax/Bcl-2 ratio and the induction of G_0_/G_1_ cell cycle arrest via inactivation of ERK1/2. Despite preliminary evidences describing the involvement of both caspase-mediated intrinsic and extrinsic pathways, further elucidation on other apoptotic targets coupled with *in vivo* studies are required for further development of this phytosterol oxide.

## Supplementary Material

A complete description of the entire extraction process and identified compounds is shown. Briefly, dried ground bark of Chisocheton tomentosus (3.5 kg) was defatted with hexane and dichloromethane for five days. The crude extract was evaporated using a rotary evaporator. The extract (10.0g) was subjected to a silica gel column and eluted gradiently using hexane:DCM and DCM:acetone. Fraction 5 of DCM:acetone (60:40) was further subjected to an isocratic separation using silica gel column with acetone:DCM:hexane (25:25:50). A colorless crystal (0.2 g) of fraction 3 was obtained from the isocratic separation.

## Figures and Tables

**Table 1:** 1D (^1^H and ^13^C) and 2D (HMQC and HMBC) NMR spectral data of of 7*α*-hydroxy-*β*-sitosterol (CT1).

Position	*δ*_H_ (int.; mult.; *J* (Hz))	*δ*_c_	HMQC	HMBC
1a	1.80 (1H, m)	37.08	H_a_–C1	1, 3, 5, 10
1b	1.01 (1H, m)		H_b_–C1	1, 3, 5, 10
2a	1.80 (1H, m)	31.3	H_a_–C2	1, 3
2b	1.47 (1H, m)		H_b_–C2	1, 3
3	3.54 (1H, m)	71.3	H–C3	
4	2.29 (2H, d, 5)	42.0	H_2_–C4	2, 5, 6, 10
5		146.3		
6	5.55 (1H, d, 5.0)	123.8	H–C6	4, 7, 8, 10
7	3.81 (1H, brs)	65.4	H–C7	5, 6, 9
8	1.43 (1H, m)	37.5	H–C8	14, 10, 4, 13, 9
9	1.15 (1H, m)	42.3	H–C9	8, 10, 11, 12, 19
10		37.4		
11	1.49 (2H, m)	20.7	H_2_–C11	9, 10, 12, 13
12a	1.97 (1H, m)	39.2	H_a_–C12	9, 13, 14
12b	1.12 (1H, m)		H_b_–C12	9, 13, 14
13		42.2		
14	1.41 (1H, m)	49.4	H–C14	15, 16, 17, 18
15a	1.66 (1H, m)	24.3	H_a_–C15	
15b	1.08 (1H, m)		H_a_–C15	
16a	1.83 (1H, m)	28.3	H_a_–C16	13, 20, 21
16b	1.22 (1H, m)		H_a_–C16	13, 20, 21
17	1.14 (1H, m)	55.7	H–C17	18, 21, 15, 16
18	0.65 (3H, s)	11.7	H_3_–C18	12, 13, 14, 17
19	0.95 (3H, s)	19.2	H_3_–C19	1, 2, 9, 10, 11
20	1.33 (1H, m)	36.1	H–C20	17, 18, 23, 24
21	0.89 (3H, d, 6.4)	18.3	H_3_–C21	17, 20, 22
22	2.24 (2H, m)	33.8	H_2_–C22	
23	1.22 (2H, m)	29.8	H_2_–C23	22, 24, 25
24	0.93 (1H, m)	49.4	H–C24	29, 26, 27
25	1.64 (1H, m)	29.0	H–C25	24, 26, 27, 28
26	0.81 (3H, m)	19.9	H_3_–C26	24, 28
27	0.77 (3H, m)	18.9	H_3_–C27	24, 25, 26
28	1.22 (2H, m)	23.1	H_2_–C28	23, 24, 25, 27, 29
29	0.83 (3H, m)	12.1	H_3_–C29	23, 24, 25, 28

**Table 2:** Summary of IC_50_ values and total cell viability of CT1-treated tumor cell lines and HMEC cells as obtained from MTT cell viability assays after 24 h exposure. All data are presented as mean ± SEM after deduction of DMSO solvent-induced cytotoxicity of three independent experiments.

Cell lines	Time (H)	IC_50_ (*μ*M)^†^	Cell viability (%)
Human mammary epithelial cell (HMEC)	24	n.d	82.4 ± 4.2
	48	n.d	80.3 ± 5.6

Human breast adenocarcinoma (MCF-7)	6	n.d	45.5 ± 5.5
	12	38.2 ± 3.2	36.5 ± 1.9
	18	28.6 ± 4.1	29.3 ± 1.4
	24	16.0 ± 3.6	21.3 ± 3.1

Human hepatocyte carcinoma (HepG2)	6	76.8 ± 5.2	35.9 ± 8.4
	12	74.0 ± 4.3	32.5 ± 9.3
	18	75.0 ± 5.1	26.8 ± 7.2
	24	25.0 ± 3.3	25.2 ± 3.6

Human oral squamous cell carcinoma (HSC-4)	6	30.3 ± 2.4	48.6 ± 1.7
	12	28.8 ± 5.1	48.1 ± 1.0
	18	24.2 ± 3.1	43.2 ± 4.3
	24	19.5 ± 2.6	40.6 ± 3.2

Human cervical carcinoma (Ca Ski)	6	n.d	94.6 ± 4.2
	12	82.9 ± 4.7	73.7 ± 7.7
	18	88.3 ± 6.3	58.8 ± 4.8
	24	96.5 ± 5.3	60.7 ± 3.6

^†^n.d denotes that total cell viability was maintained >50% viability at maximum incubation time and CT1 concentration.
