# Supplementary material for: 7α-Hydroxy-β-Sitosterol from Chisocheton tomentosus Induces Apoptosis via Dysregulation of Cellular Bax/Bcl-2 Ratio and Cell Cycle Arrest by Downregulating ERK1/2 Activation
Source: Evid Based Complement Alternat Med. 2012 Sep 11;2012:765316. doi: 10.1155/2012/765316 (PMC3446807; doi:10.1155/2012/765316)
Supplement: Supplementary file 1 — A complete description of the entire extraction process and identified compounds is shown. Briefly, dried ground bark of Chisocheton tomentosus (3.5 kg) was defatted with hexane and dichloromethane for five days. The crude extract was evaporated using a rotary evaporator. The extract (10.0g) was subjected to a silica gel column and eluted gradiently using hexane:DCM and DCM:acetone. Fraction 5 of DCM:acetone (60:40) was further subjected to an isocratic separation using silica gel column with acetone:DCM:hexane (25:25:50). A colorless crystal (0.2 g) of fraction 3 was obtained from the isocratic separation. [file 765316.f1.docx]

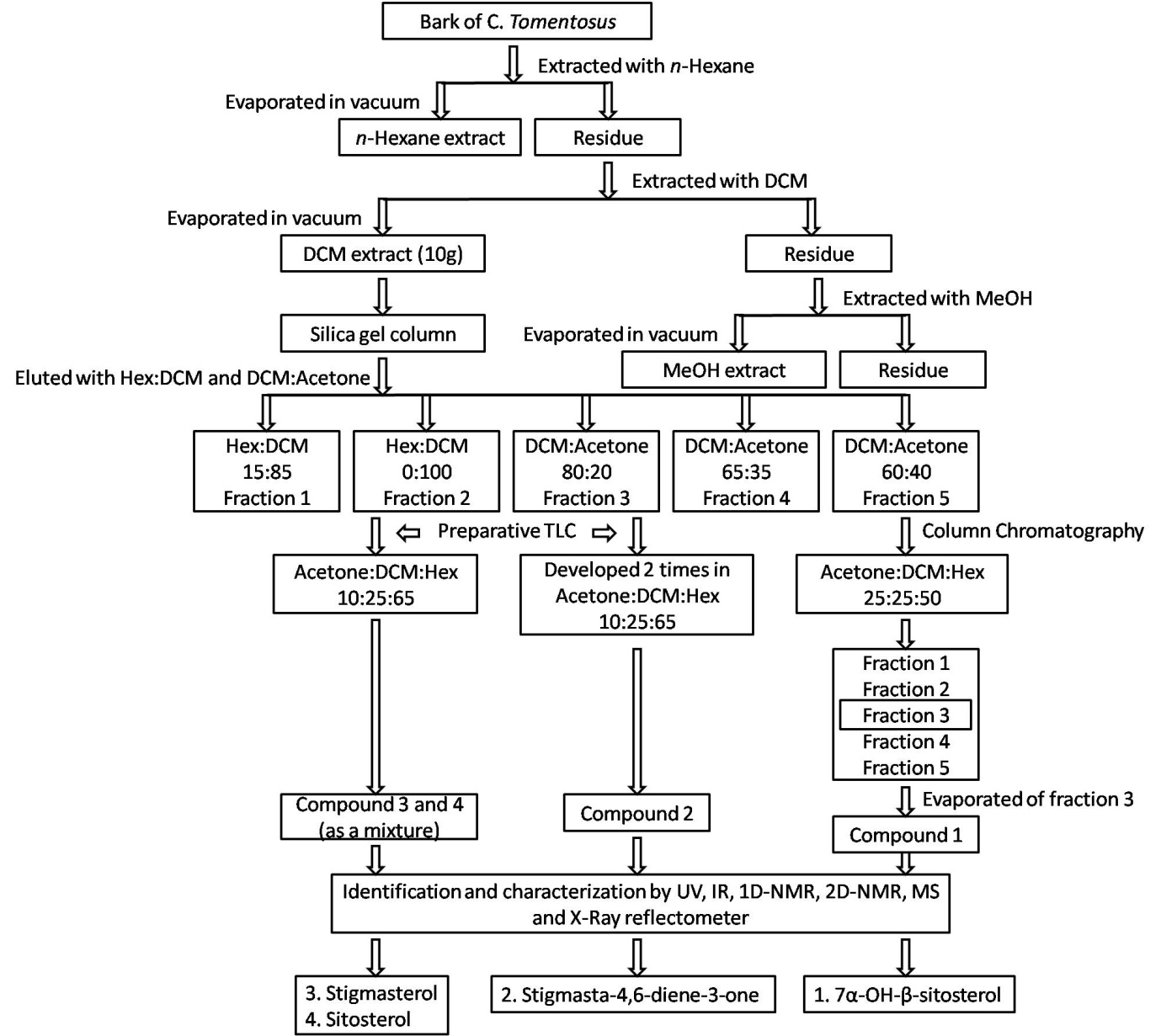


**Supplementary Material 1**: Schematic diagram describing the entire extraction process and compounds identified. Adapted from [31].
